# Induction of Osmolyte Pathways in Skeletal Muscle Inflammation: Novel Biomarkers for Myositis

**DOI:** 10.3389/fneur.2018.00846

**Published:** 2018-10-11

**Authors:** Boel De Paepe, Jana Zschüntzsch, Tea Šokčević, Joachim Weis, Jens Schmidt, Jan L. De Bleecker

**Affiliations:** ^1^Department of Neurology and Neuromuscular Reference Center, Ghent University Hospital, Ghent, Belgium; ^2^Department of Neurology, University Medical Center Göttingen, Göttingen, Germany; ^3^Institute for Neuropathology, Reinisch-Westfälische Technische Hochschule Aachen University Hospital, Aachen, Germany

**Keywords:** inflammatory myopathy, osmotic stress, inflammatory stress, osmolytes, muscle regeneration

## Abstract

We recently identified osmolyte accumulators as novel biomarkers for chronic skeletal muscle inflammation and weakness, but their precise involvement in inflammatory myopathies remains elusive. In the current study, we demonstrate *in vitro* that, in myoblasts and myotubes exposed to pro-inflammatory cytokines or increased salt concentration, mRNA levels of the osmolyte carriers SLC5A3, SLC6A6, SLC6A12, and AKR1B1 enzyme can be upregulated. Induction of SLC6A12 and AKR1B1 was confirmed at the protein level using immunofluorescence and Western blotting. Gene silencing by specific siRNAs revealed that these factors were vital for muscle cells under hyperosmotic conditions. Pro-inflammatory cytokines activated mitogen-activated protein kinases, nuclear factor κB as well as nuclear factor of activated T-cells 5 mRNA expression. In muscle biopsies from patients with polymyositis or sporadic inclusion body myositis, osmolyte pathway activation was observed in regenerating muscle fibers. In addition, the osmolyte carriers SLC5A3 and SLC6A12 localized to subsets of immune cells, most notably to the endomysial macrophages and T-cells. Collectively, this study unveiled that muscle cells respond to osmotic and inflammatory stress by osmolyte pathway activation, likely orchestrating general protection of the tissue. Moreover, pro-inflammatory properties are attributed to SLC5A3 and SLC6A12 in auto-aggressive macrophages and T-cells in inflamed skeletal muscle.

## Introduction

The idiopathic inflammatory myopathies represent a diverse group of autoimmune muscle diseases. The main disease entities recognized today are dermatomyositis (DM), polymyositis (PM), sporadic inclusion body myositis (IBM), immune-mediated necrotizing myopathy (IMNM), anti-synthetase syndrome, and unspecific myositis, each of which possess distinct clinical and myopathological characteristics (1–5). DM patients develop complement-mediated blood vessel destruction, perimysial inflammation and perifascicular muscle fiber atrophy (6). PM and IBM are characterized by invasion of nonnecrotic muscle fibers by auto-aggressive cytotoxic T-cells and macrophages, with inflammation building up mostly at endomysial sites (7). In IBM muscle fibers, additional degenerative phenomena occur, with rimmed vacuoles and inclusions that contain aggregates of ectopic proteins (8), a process that is presumed to follow inflammation (9). IMNM represents 3 subdivisions according to serologic characteristics: anti-3-hydroxy-3-methylglutaryl-coenzyme A reductase myopathy, signal recognition particle myopathy, and myositis-specific autoantibody negative IMNM (10). Many of the immunopathogenic mechanisms underlying the inflammatory myopathies remain poorly understood, hampering the development of successful therapies that suit the different patient subtypes.

Muscle is a highly adaptive and dynamic tissue capable of increasing its mass in response to exercise and of restoring damage caused by injury via processes that require hypertrophy and regeneration, respectively. In addition, cells possess a universal ability to adapt to changing osmotic conditions, a feature essential for their survival, allowing active anticipation toward perturbations in volume and disrupted cellular homeostasis. A complex intracellular mixture of interacting osmolytes regulates osmotic pressure, which takes shape through the synthesis and/or import of osmo-active compounds in cells (11). The solute carrier family (SLC) contains several salt-dependent membrane transport proteins involved in the selective import of extracellular constituents: (i) the sodium-myoinositol cotransporter SLC5A3, (ii) the high-affinity taurine transporter SLC6A6, and (iii) the betaine and γ-amino-n-butyric acid (GABA) transporter SLC6A12. AKR1B1 is an aldose reductase that catalyzes the intracellular conversion of glucose to sorbitol. In a joint effort, these osmoprotective constituents represent a universal tool for mammalian cells, and their accumulators can be ubiquitously expressed throughout human tissues. The central regulator of osmolyte pathway gene expression is the transcription factor nuclear factor of activated T-cells 5 (NFAT5), also termed tonicity enhancer-binding protein (12).

Several observations allowed us to speculate NFAT5-inducible pathways might be involved in the inflammatory myopathies. Firstly, the NFAT5 pathway participates in muscle development and regeneration, by regulating the differentiation of immature myoblasts to mature multinucleated myotubes (13). NFAT5 levels have been shown to increase in the regenerating fibers of mice exposed to experimental muscle tissue injury (14), attributing the transcription factor a role in countering disease-inflicted muscle tissue damage. Secondly, the NFAT5 pathway has been firmly linked to nuclear factor κB (NFκB) activity (15), the latter a key regulator of inflammatory diseases in general and the inflammatory myopathies in particular (16). NFAT5 and NFκB share multiple molecular targets (17), many of which are involved in the immunopathogeneses of inflammatory myopathy including CCL2, also termed monocyte chemo-attractant protein-1 (MCP-1) (18), lymphotoxin β (LTβ) (19), tumor necrosis factor α (TNFα) (20), inducible nitric oxide synthase (iNOS) (21), and heat shock protein family of 70kd (HSP70) (22). Thirdly, NFAT5-downstream osmolyte pathways are potent activators of cytotoxic activities of immune cells and could therefore be implicated in human autoimmune disease. In addition, dietary salt is determinant to T-cell differentiation, by direct activation of glycogen synthase kinase 1 (GSK-1) and subsequent Interleukin 23 receptor stabilization, which enforces the type 17 helper T-cell (Th17), a T-cell phenotype associated with autoimmune disease (23).

We recently were the first to describe upregulation of the osmolyte accumulators SLC5A3, SLC6A6 and AKR1B1 in muscle tissues from myositis patients (24), and described NFAT5 expression in myoblasts in culture (25), yet the precise role osmolyte pathways play in disease mechanisms remained unexplored. With this study, we aim to substantiate and functionally connect these biomarkers with muscle inflammation. We set up *in vitro* muscle cell models to investigate osmolyte pathway member expression, using both the human rhabdomyosarcoma CCL-136 cell line and normal primary human myotubes. In addition, we investigated their possible signaling routes, which involves the upstream transcription factors NFκB and NFAT5, and the mitogen-activated protein kinases (MAPKs). We confronted this *in vitro* evidence with findings in muscle biopsies from patients diagnosed with inflammatory myopathies.

## Methods

### Cell cultures

Human rhabdomyosarcoma CCL-136 cells (ATCC, Manassas, VA) were kept in Dulbecco's modified Eagle medium (DMEM) supplemented with 10% fetal calf serum (Biochrom, Berlin, Germany) and 1% L-glutamine (ThermoFisher Scientific, Waltham, MA). Human muscle cell cultures originated from muscle biopsies taken from healthy patients needing knee surgery, obtained with patient consent and approved by the local ethics committee. Biopsies were minced and trypsinized. Fragments were seeded in DMEM with pyruvate, high glucose and L-glutamine, supplemented with penicillin, streptomycin and 10% fetal calf serum (ThermoFisher Scientific). Cells were cultured for 2 periods of ~ 3 weeks during which CD56+ cells were purified twice with magnetic separation MiniMACS columns using the supplier's standard protocol (Miltenyi Biotec, Bergisch Gladbach, Germany). Myotubes were obtained by allowing cells to differentiate for approximately 5 days in DMEM medium supplemented with penicillin, streptomycin and 2% horse serum (ThermoFisher Scientific). Supplementary Figure S1 illustrates a representative culture of differentiated myotubes, showing aligned multinucleate muscle cells. All cells were kept in culture wells or chamber slides at 37°C in a humified atmosphere containing 5% CO_2_. Conditions for cytokine stimulation were as determined earlier (26), being 30 ng/ml TNFα, 300 u/ml Interferon γ (IFNγ), 20 ng/ml Interleukin 1β (IL1β) (R and D Systems, Minneapolis, MN), or double combinations. Hyperosmotic conditions were created by supplementing the culture medium with 25–150 mM of added NaCl. Higher concentrations of NaCl lead to complete cell death within 24 h.

### Quantitative reverse transcription PCR

RNA was prepared from cells cultured in 12-well culture plates, using the RNeasy Mini kit and according to the manufacturer's specifications (Qiagen, Hilden, Germany). RNA concentration was measured with a Nanodrop 1000 (ThermoFisher Scientific). cDNA was prepared from 200 ng of RNA with SuperScript II reverse transcriptase, 500 ng/μl oligo dTs, 0.1 M DTT, and 10 mM dNTPs each (Invitrogen, Darmstadt, Germany). cDNA was quantified through PCR reaction with Taqman Gene Expression Master mix (Applied Biosystems, Foster City, CA), using 6-carboxy-fluorescein-labeled probes and specific primers for: SLC5A3, Hs00272857_s1; SLC6A6, Hs00161778_m1; SLC6A12, HS00758246_m1; AKR1B1, Hs00739326_m1; NFAT5, Hs00232437_m1; MAPK14, Hs01051152_m1; RELA, Hs00153294_m1; NFKB1, Hs00765730_m1; NFKB2, Hs01028901_g1; glyceraldehyde-3-phosphate dehydrogenase (GAPDH), Hs99999905_m1 (Applied Biosystems). Reactions were run in triplicate, following the standard cycle protocol on a 7500 Real Time PCR System, and analyzed with software version 2.0.6. (Applied Biosystems). Data was presented as ΔΔCt fold-changes compared to the expression levels in untreated cells, with GAPDH as an internal housekeeping gene standard.

### Immunofluorescent cytostaining

Cells cultured on glass chamber slides were fixed with ice-cold acetone and blocked in phosphate buffered saline with 10% bovine serum albumin and 10% goat serum for 1 h at room temperature. Immunofluorescent detection was carried out for 1 h at room temperature with commercially available antibodies: mouse (4 μg/ml, sc-514024, SantaCruz Biotechnology, Santa Cruz, CA) and rabbit (10 μg/ml, nbp188641, Novus Biologicals, Abingdon, UK) anti-SLC6A12; rabbit (2 μg/ml, sc-33219, SantaCruz Biotechnology) and goat (1 μg/ml, sc-17732, SantaCruz Biotechnology) anti-AKR1B1; mouse anti-developmental myosin heavy chain (dMyHC) (40 μg/ml, RMMy2/9D2, Leica Biosystems, Nussloch, Germany). The corresponding Alexa594—and Alexa488-labeled secondary antibodies (ThermoFisher Scientific) were added. After mounting in Fluoromount G (Southern Biotech, Alabama, USA), digital photography was performed on a Zeiss Axiophot microscope (Zeiss, Goettingen, Germany). Pictures were taken by a cooled CCD digital camera (Retiga 1300, Qimaging, Burnaby, BC, Canada) and visualized with ImageProPlus software (MediaCybernetics, Bethesda, MD).

### Immunofluorescent histostaining

For localization studies in muscle tissues, 8 μm cryostat sections were cut from frozen muscle biopsies obtained from patients without muscle abnormalities (*n* = 10), inflammatory myopathies (*n* = 28), and disease controls diagnosed with muscular dystrophy (*n* = 8; for pathological information consult Supplementary Table S1). Diagnosis of the disease subgroups PM (*n* = 4), IBM (*n* = 9), DM (*n* = 9), and IMNM (*n* = 6) were based upon clinical and myopathological criteria (27). Diagnosis of PM was reserved to patients with nonnecrotic invaded muscle fibers present in the biopsy that had subsequently responded to immunosuppressive therapy. Sections were fixed in ice-cold acetone and treated with blocking solution containing 5% donkey serum, 10% heat-inactivated human serum and 2% bovine serum albumine in phosphate buffered saline. Incubations with primary antibodies were carried out in the same solution: 4 μg/ml rabbit polyclonal anti-SLC5A3 (NBP1-02399, Novusbio); 4 μg/ml mouse monoclonal anti-SLC6A6 (E10, SantaCruz Biotechnology); 6 μg/ml rabbit polyclonal anti-SLC6A12 (HPA034973; Merck, Kenilworth, NJ); 1 μg/ml goat polyclonal anti-AKR1B1 (N20, SantaCruz Biotechnology). Immune cell subtypes and muscle tissue constituents were visualized through double staining as described (18). Satellite cells were visualized with goat anti-Pax3/7 (1 μg/ml, sc-7748, SantaCruz Biotechnology). To allow double staining of macrophages with mouse monoclonal antibodies, FITC labeled anti-CD68 (Agilent, Santa Clara, CA) was applied. Secondary antibodies were used labeled with CY3 (Jackson ImmunoResearch Laboratories, West Grove, PA) and AlexaFluor488 (ThermoFisher Scientific). Slides were mounted with Fluoromount (Southern Biotech) and visualized with a fluorescence microscope (Zeiss). Conventional semi-quantitative scoring of staining intensity was performed by three non-blinded independent observers. Negative control studies consisted of the omission of primary antibody and the substitution by non-immune IgGs. Positive control tissues for checking immunodetection were cultured Hela cells (SLC6A6), frozen sections containing kidney medulla (SLC5A3, SLC6A12), and Jurkat cells (AKR1B1).

### Western blotting

Cells cultured in 12-well culture plates were lysed in Ripa buffer (50 mM Tris-HCl, 150 mM NaCl, 2.5% Na-deoxycholate, 2.5% NP40, 0.1% sodium dodecyl sulfate pH 7.4) with a protease inhibitor cocktail added (Roche, Indianapolis, IN) and centrifuged for 5 min at 13000 rpm. The supernatant was collected and protein concentrations were determined following a Bradford procedure (Bio-Rad protein assay, Hercules, CA) with bovine serum albumine standard solutions, measured in triplicates on the Infinite M200Pro and analyzed with Magellan 7.2 software (Tecan, Mannedorf, Switzerland). 60 μg protein samples were dissolved in Laemli buffer, boiled for 2 min, separated by 12% sodium dodecyl sulfate-polyacrylamide gelelectrophoresis, and transferred to a nitrocellulose membrane (Schleicher and Schuell, Dassel, Germany). Membranes were blocked with 5% bovine serum albumine for 1 h at 4°C and incubated overnight at 4°C with 2 μg/ml mouse anti-SLC6A12 (sc-514024, SantaCruz Biotechnology), 4 h at room temperature with 2 μg/ml goat anti-AKR1B1 (sc-17732, SantaCruz Biotechnology), and 1 h at room temperature with 0.7 μg/ml mouse anti-GAPDH (Sp210-Ag14; Abcam, Cambridge, UK). All incubations were done in tris-buffered saline buffer containing 0.05% Tween20. Appropriate horseradish peroxidase-conjugated secondary antibodies (Jackson ImmunoResearch, West Grove, PA) were added for 1 h at room temperature. The chemiluminescent signal was generated with the Pierce Western blotting substrate (ThermoFisher Scientific), visualized with the Fusion FX, and quantified with Vision Capt software, with background noise filtering using a rolling-ball algorithm (Vilber Lourmat, Eberhardzell, Germany).

### Protein phosphorylation profiling

Protein phosphorylation patterns were determined in extracts obtained from cultured myotubes, using the Proteome Profiler human phosphor-mitogen activated protein kinase (MAPK) antibody array according to the manufacturer's specifications (Bio-Techne, Abingdon, United Kingdom). Briefly, array membranes were incubated overnight at 4°C with lysate containing 200 μg of cellular protein, detection conditions were as described in the western blotting section. Protein densities were quantified, relative to phosphorylated Akt2, as the calculated mean of duplicate spots per protein, using Image Studio 5.2 software (Li-Cor Biosciences, Cambridge, UK).

### Knockdown studies

Pools of three target-specific 19–25 nucleotide silencing RNAs (siRNAs), purchased from SantaCruz Biotechnology, were used: siRNA SLS5A3 (sc-44516), siRNA SLC6A12 (sc-95904), and siRNA AKR1B1 (sc-37119). 50% confluent CCL-136 cells and myotube cultures were changed to 500 μl X-Vivo15 medium (Sartorius, Goettingen, Germany), to which 3 μl of lipofectamine (ThermoFisher) and 100 nM siRNA had been added. After 5 h, an extra 200 μl of X-Vivo15 was added, which in treated cells contained cytokines to a final well concentration of 20 ng/ml IL1β+300 u/ml IFNγ, or 50 mM added NaCl. Cells were assayed 26 h after addition of the siRNAs. Live and dead cells were visualized using the ReadyProbes cell viability imaging blue/green kit (ThermoFisher) according to the manufacturer's specifications. Using a fluorescence microscope, a minimum of 50 cells (blue) was counted per condition, determining the amount of dead cells (green). Afterwards, cell cultures were stained with hematoxylin and eosin according to standard procedures, dehydrated, mounted, and interpreted under a light microscope. Efficiency of knockdown was evaluated at the mRNA level using quantitative reverse transcription PCR, following the method described above. From controls and siRNA-treated CCL-136 cells seeded in 12-well culture plates, protein samples were prepared for electrophoresis by adding lithium dodecyl sulfate sample buffer and reducing agent (Invitrogen, Carlsbad, CA, USA) and boiling for 3 min. Samples were loaded onto 10% bis-tris gels, with prestained markers alongside to determine the molecular weight of protein bands. Proteins were transferred to nitrocellulose membranes by electroblotting, and incubated with 2 μg/ml mouse anti-SLC6A12 (sc-514024), 2 μg/ml goat anti-AKR1B1 (sc-17732), or mouse anti-β actin (sc-47778) (SantaCruz Biotechnology) for 4 h at room temperature on a rocking platform. Immunoreaction was visualized using the chromogenic Western Breeze kit according to the manufacturer's specifications (Invitrogen).

### Compliance with ethical standards

Human experimentation presented in the study was approved by the Ghent University Hospital Ethics Committee (EC-UZG-#B670201316956) and adhered to privacy regulations (CBPL-BEL-#HM003039095). Written informed consent was obtained from all individual participants included in the study, and all procedrues were in accordance with the Declaration of Helsinki.

## Results

### mRNA quantification in cultured muscle cells

In CCL-136 cells, *e*xpression levels of osmolyte pathway members were determined in cells treated with pro-inflammatory cytokines or added NaCl for 24 h (Table 1). In general, a moderate increase of expression was shown for SLC5A3, SLC6A6, SLC6A12, and AKR1B1 when cells were treated with cytokines. The strongest response was a 10-fold increase of SLC5A3 mRNA expression in IL1β+TNFα-treated cells. Hyperosmotic conditions also induced osmolyte pathway expression, with 100 mM of added NaCl leading to a 179-fold increase of SLC6A12, and increasing NFAT5 expression 2.5-fold. MAPK14 levels were 2-fold increased in both IFNγ+TNFα– and 50 mM NaCl-treated cells. Highest levels of RelA, NFkB1 and NFkB2 could be achieved with pro-inflammatory cytokine mixtures, though NFkB1 and NFkB2 expression also responded to added NaCl.

**Table 1:** Messenger RNA levels in cultured CCL-136 cells treated for 24 h.

	**Cytokines**						**Added NaCl**			
	**IFNγ**	**IL1β**	**TNFα**	**IFNγ+IL1β**	**IFNγ+TNFα**	**IL1β+TNFα**	**25 mM**	**50 mM**	**75 mM**	**100 mM**
SLC5A3	1	1.1	1.3	2.1	4.6	10.3	0.6	0.5	1.9	3.4
SLC6A6	0.5	0.7	0.8	1.4	1.5	4.3	0.9	1.1	1.2	1.5
SLC6A12	1.7	2	1	3.4	3.8	1.2	1.6	7.7	9	178.7
AKR1B1	1.4	2.3	1.9	1.9	1.6	1.7	1.4	2.1	2.4	5.6
NFAT5	1.1	0.3	0.5	1.4	1.7	1	1.4	4.1	0.8	2.5
MAPK14	0.7	0.8	0.8	1.4	1.9	1.4	1.1	2	0.9	1.3
RELA	0.9	1.2	0.9	1.8	1.7	0.5	0.9	0.8	0.9	1.2
NFkB1	1	1.2	1	6	0.5	3.8	1.5	2.9	1.2	1.9
NFkB2	1.3	1.5	2.7	3.3	12.9	1	1.2	2.3	1.5	1.1

*Fold changes compared to untreated cells, normalized to glyceraldehyde 3-phosphate dehydrogenase expression levels, were obtained through quantitative reverse transcription PCR and calculated as 2^−ΔΔCt^ (mean of three given). Fold changes >2 green; >5 yellow; >10 orange; >100 red*.

Primary human myotubes treated for 24 h with cytokines or added NaCl showed induction of osmolyte pathway mRNA levels (Table 2). For SLC5A3 and AKR1B1, the strongest induction could be achieved with added NaCl; SLC6A6 and SLC6A12 were most strongly induced by pro-inflammatory cytokine mixtures. NFAT5 expression was influenced by both cytokines and increased salt concentrations, but reached higher expression levels with IFNγ+IL1β (4,5-fold) than with 125 mM NaCl (3-fold). MAPK14 expression levels were found increased 2-fold in myotubes treated with IL1β and with 125 mM NaCl. The expression of NFκB subunits was most strongly induced by treatment with pro-inflammatory cytokines. Nonetheless, 25 mM added NaCl increased NFkB1 expression 8,5-fold. In myotubes treated for longer periods, added NaCl lead to continuously increasing SLC5A3, SLC6A12 and AKR1B1 expression levels over time, culminating in levels all exceeding 200-fold at time point 72 h (Table 3). In addition, prolonged salt treatment lead to a time-dependent increase of NFAT5 and MAPK14 expression levels, reaching a maximum of 9-fold (NFAT5) and 18-fold (MAPK14). In contrast, the single pulse of pro-inflammatory cytokines resulted in highest expression levels after 24 h, with levels nearing normal after 72 h.

**Table 2:** Messenger RNA levels in cultured normal human myotubes treated for 24 h.

	**Cytokines**						**Added NaCl**					
	**IFNγ**	**IL1β**	**TNFα**	**IFNγ+IL1β**	**IFNγ+TNFα**	**IL1β+TNFα**	**25 mM**	**50 mM**	**75 mM**	**100 mM**	**125 mM**	**150 mM**
SLC5A3	2.6	5.8	2.5	2.4	1.4	1.9	1.4	2.1	3.6	13	13.9	5
SLC6A6	1.2	1.9	2.9	3.9	2.2	3.1	1.1	1.9	1.8	1.9	1.8	0.4
SLC6A12	21.3	2.1	1.4	177.7	229.2	3.8	2.1	5.9	7.3	32.4	47.9	9.6
AKR1B1	1.5	6.6	3.7	7.5	5.6	4.9	10.7	3.5	4.7	6.9	8.1	2.5
NFAT5	1.4	4.4	1.6	4.5	2.2	2	0.8	1.6	1.3	2.2	3	0.6
MAPK14	1	1.9	1.1	0.6	0.4	0.9	1.4	1.3	1	1.6	1.9	0.6
RELA	2.1	2	1.4	25.3	18.4	4.2	0.8	1.1	1.2	1.1	1.2	0.5
NFkB1	1.5	1.3	0.8	6.8	3.6	2.6	8.7	0.7	0.7	0.6	1.3	0.7
NFkB2	1.2	4.7	2.7	20.5	4	8.4	0.1	1.3	1.2	0.8	1	0.3

*Fold changes compared to untreated cells, normalized to glyceraldehyde 3-phosphate dehydrogenase expression levels, were obtained through quantitative reverse transcription PCR and calculated as 2^−ΔΔCt^ (mean of three given). Fold changes >2 green; >5 yellow; >10 orange; >100 red*.

**Table 3:** Messenger RNA levels in cultured normal human myotubes treated with cytokines or added NaCl for up to 3 days.

	**IFN**γ+**IL1**β			**IFN**γ+**TNF**α			**100 mM NaCl**		
	**24 h**	**48 h**	**72 h**	**24 h**	**48 h**	**72 h**	**24 h**	**48 h**	**72 h**
SLC5A3	1	0.4	0.2	1	0.6	0.5	10.8	5.8	240.7
SLC6A12	19.1	4.9	1	14.5	13	2.5	43	50.9	2 × 10exp8
AKR1B1	2.4	1.3	0.9	1.7	1.9	1.1	7	27.4	528.8
NFAT5	0.8	0.3	0.04	0.8	0.9	0.3	1.4	3.8	9.4
MAPK14	0.9	0.4	0.3	0.6	0.6	0.6	1.1	1.6	17.8

*Fold changes compared to untreated cells, normalized to glyceraldehyde 3-phosphate dehydrogenase expression levels, were obtained through quantitative reverse transcription PCR and calculated as 2^−ΔΔCt^ (mean of three given). Fold changes >2 green; >5 yellow; >10 orange; >100 red*.

### Immunofluorescent protein localization studies in cultured muscle cells

**Figure 1:**
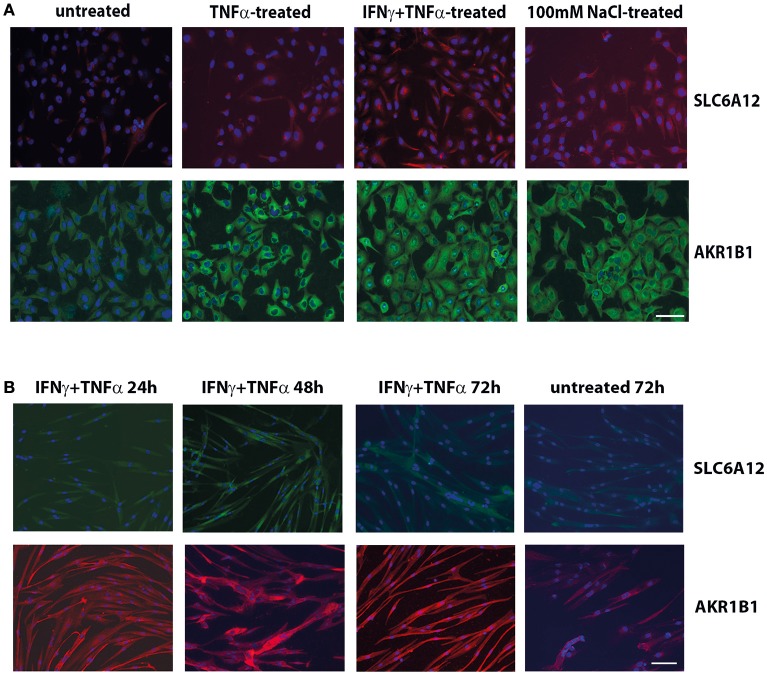
Immunofluorescent cytostaining. **(A)** Staining with mouse anti-SLC6A12 (AlexaFluor 594, red) and rabbit anti-AKR1B1 (AlexaFluor488, green) in CCL-136 cells after 24 h treatment. Untreated control cells shows low levels of SLC6A12 and AKR1B1. Treatment with 30 ng/ml TNFα markedly increases AKR1B1 levels. Treatment with 300 u/ml IFNγ and 30 ng/ml TNFα strongly increases both SLC6A12 levels and AKR1B1 protein levels. Addition of 100 mM NaCl to the medium also increases both SLC6A12 and AKR1B1 protein expression. **(B)** Staining with rabbit anti-SLC6A12 (AlexaFluor 488, green) and goat anti-AKR1B1 (AlexaFluor594, red) in cultured healthy human myotubes at different time points. Myotubes treated with 300 u/ml IFNγ and 30 ng/ml TNFα display low levels of SLC6A12 that is increased at the 48 h time point. Levels return back to constitutive low levels after 72 h, with staining levels similar to those in untreated cells. Staining for AKR1B1 shows continuously high levels between 24 and 72 h. In untreated cells, AKR1B1 expression levels are substantially lower. Scale bar = 50 μm.

Immunofluorescent staining of CCL-136 cells (Figure 1A) confirmed the induction of SLC6A12 and AKR1B1 expression after 24 h of treatment with pro-inflammatory cytokines and added NaCl at the protein level and showed similar staining patterns with the two different sets of primary antibodies.

Myotubes immunostaining (Figure 1B) showed increases of SLC6A12 in response to cytokines at the 48 h time point, with levels declining again at time point 72 h. For AKR1B1, high levels were detected following cytokine stimulation from 24 h onward, compared to the low levels observed in untreated myotubes. A growth-inhibitory effect of hyperosmotic conditions was observed in myotubes, which became conspicuous from the 48 h time point on. NaCl-treatment disfavored differentiation into multinucleate elongated muscle cells, as was assayed with dMyHC staining (Supplementary Figure S1). Evaluation of hematoxylin&eosin stains confirmed that salt treatment affected cell morphology and growth, reducing cell elongation and disfavoring multinucleate myotubes.

### Protein quantification and phosphorylation patterns of human myotubes

**Figure 2:**
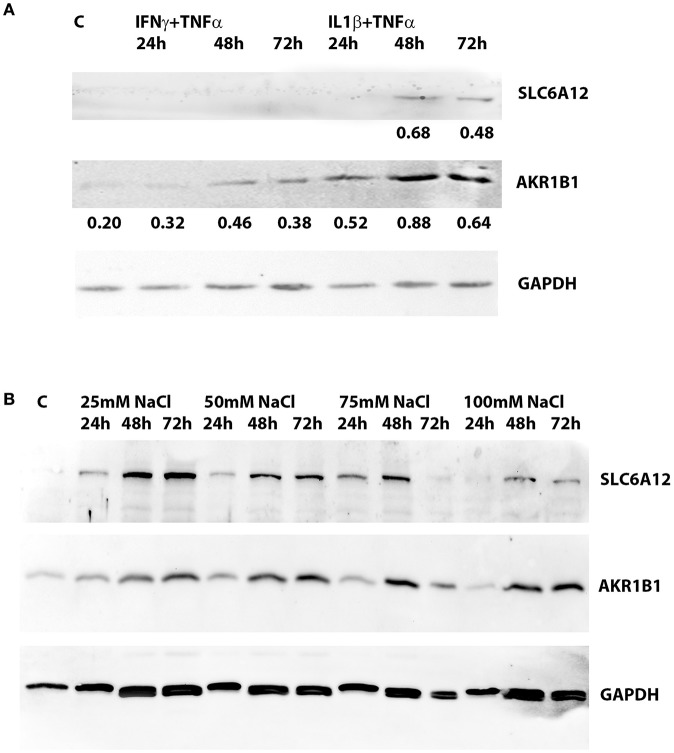
Western blotting of protein extracts preepared from cultured normal human myotubes. **(A)** SLC6A12 and AKR1B1 protein levels in myotubes treated with cytokine mixtures. SLC6A12 and AKR1B1 proteins are visualized in control cells (C) and in cells treated for 24 h-48 h-72 h with either 300 u/ml IFNγ+30 ng/ml TNFα, or 20 ng/ml IL1β+30 ng/ml TNFα. SLC6A12 is undetectable in untreated- and in 300 u/ml IFNγ+30 ng/ml TNFα-treated cells, but is induced by treatment with 20 ng/ml IL1β+30 ng/ml TNFα from the 48 h time point on. Low constitutive levels of AKR1B1 in control cells are increased in myotubes treated with both cytokine mixtures, peaking at the 48 h time point. Relative protein densities of AKR1B1, normalized using glyceraldehyde 3-phosphate dehydrogenase (GAPDH) levels as an internal standard, have been indicated. **(B)** SLC6A12 and AKR1B1 protein levels in myotubes treated with added NaCl. Protein bands for SLC6A12 and AKR1B1, and the internal standard glyceraldehyde 3-phosphate dehydrogenase (GAPDH), are given in an untreated control (C) and in cells treated with different concentrations of added NaCl. A time-dependent increase is observed in cells treated with 25 and 50 mM added NaCl, while more elevated NaCl concentrations show the highest levels at the 48 h timepoint. The corresponding relative protein densities are listed in Table 4.

SLC6A12 protein levels were below the detection limit in untreated normal myotubes, but SLC6A12 protein could readily be shown in myotubes treated with IL1β+TNFα for 48 h and 72 h (Figure 2A). SLC6A12 and AKR1B1 protein levels were found to increase in a time- and dose-dependent manner when myotubes were exposed to 25 and 50 mM of added NaCl (Figure 2B). The expression levels with the higher doses of NaCl already reached a maximum at the 48 h time-point (Table 4).

**Table 4:** Protein densities in cultured normal human myotubes treated with added NaCl for varying periods of time.

	**Untreated**	**25 mM NaCl**			**50 mM NaCl**			**75 mM NaCl**			**100 mM NaCl**		
		**24 h**	**48 h**	**72 h**	**24 h**	**48 h**	**72 h**	**24 h**	**48 h**	**72 h**	**24 h**	**48 h**	**72 h**
SLC6A12	0.001	0.097	0.264	**0.297**	0.139	0.180	**0.198**	0.154	**0.217**	0.131	0.112	**0.115**	0.094
AKR1B1	0.192	0.202	0.210	**0.347**	0.336	0.377	**0.437**	0.281	**0.436**	0.347	0.209	**0.278**	0.263

*Protein densities of western blots (shown in Figure 2) were normalized using glyceraldehyde 3-phosphate dehydrogenase levels. Highest protein expression levels per time point have been highlighted*.

Protein phosphorylation patterns of MAPKs showed that the strongest signals in the array were the phosphorylated forms of Akt2 (S474) and heat shock protein 27 (HSP27) (S78, S82) (Supplementary Figure S2). Akt2 and HSP27 activation was prominent in untreated and cytokine-treated myotubes alike. The most notable influence of 24 h IFNγ+IL1β treatment was an increase in phosphorylation of mitogen- and stress-activated kinase 2 (MSK2) (S360) 3-fold and MAPK3 (T202, Y204) 2.5-fold. In addition, levels of phosphorylated glycogen synthase kinase 3α/β (GSK-3α/β) (S9, S21), MAPK12 (T183, Y185), and MAPK13 (T180, Y182) were increased 2-fold.

### Knockdown studies in cultured muscle cells

**Figure 3:**
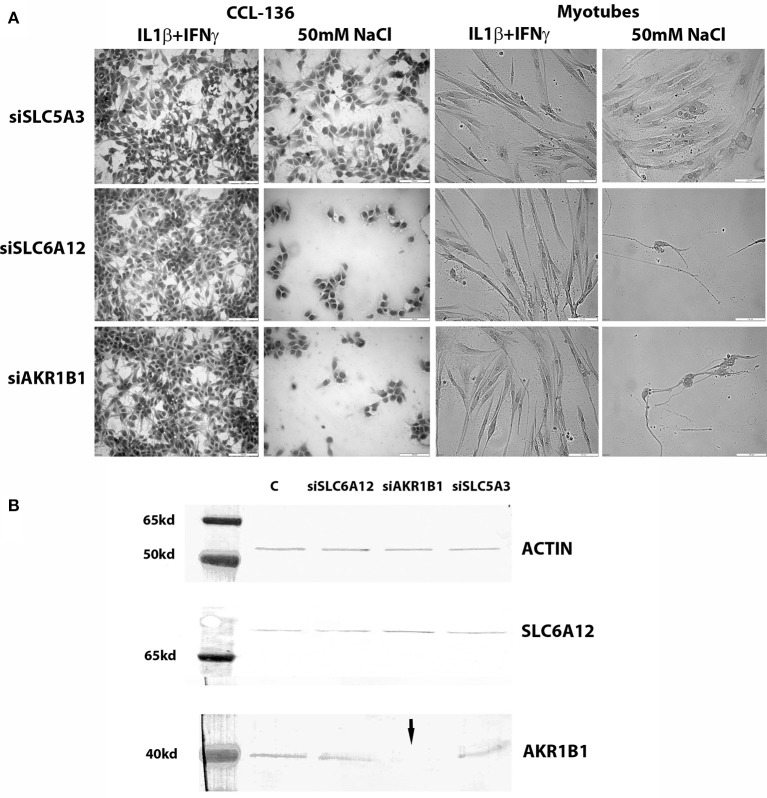
Knockdown studies. **(A)** Hematoxylin&Eosin staining of CCL-136 and healthy human myotube cultures treated with silencing RNAs. Representative images are shown of cell cultures exposed to 300 u/ml IFNγ+20 ng/ml IL1β or to 50 mM of added NaCl. Culture densities decrease substantially in siSLC6A12- and siAKR1B1-treated cultures challenged with 50 mM of added NaCl, both in CCL-136 cells and in normal human myotubes. Addition of siSLC5A3 does not apparently inhibit cell growth, only resulting in mildly reduced densities of CCL-136 cells which could equally be observed in controls treated with 50 mM NaCl. Scale bar = 100 μm. **(B)** Western blots visualizing SLC6A12 and AKR1B1 protein in CCL-136 cells treated with silencing RNAs. The severe reduction of the AKR1B1 protein band in siAKR1B1-treated cells (arrow) shows the efficiency of expression knockdown. C represents the control sample, and equal loading is shown by β-actin protein bands.

Both in CCL-136 cells and in primary healthy myotubes, 50 mM of added NaCl decreased cell densities in both siSCL6A12—and siAKR1B1-treated cells at the 24 h time point, while this regimen did not harm growth of siSLC5A3-treated muscle cells (Figure 3A). Treatment with pro-inflammatory cytokines on the other hand, combined with knockdown of individual osmolyte pathway members, did not cause significant effects on myotube viability, with percentages of dead cells in untreated vs. cytokine-treated cells respectively: 1% vs. 7% (vehicle), 11% vs. 25% (siSLC6A12), 33% vs. 15% (siAKR1B1), and 0% vs. 4% (siSLC5A3). For silencing procedures, efficiency of knockdown was evaluated in CCL-136 cells (calculated as ΔCt siRNA-treated cells minus ΔCt of the vehicle control) detecting 16% (siSLC5A3), 59% (siSLC6A12), and 6% (siAKR1B1) of residual mRNA expression. Western blotting corroborated efficient AKR1B1 knockdown at the protein level but did not show a substantial reduction of SLC6A12 protein levels (Figure 3B). We could not quantify SLC5A3 protein in muscle samples using western blots, as we were unsuccessful in detecting SLC5A3 in the corresponding positive control samples using rabbit (nbp102399, NovusBiologicals) and goat (sc-23142, SantaCruz Biotechnology) antibodies.

### Immunolocalization studies in muscle tissues from inflammatory myopathy patients

**Figure 4:**
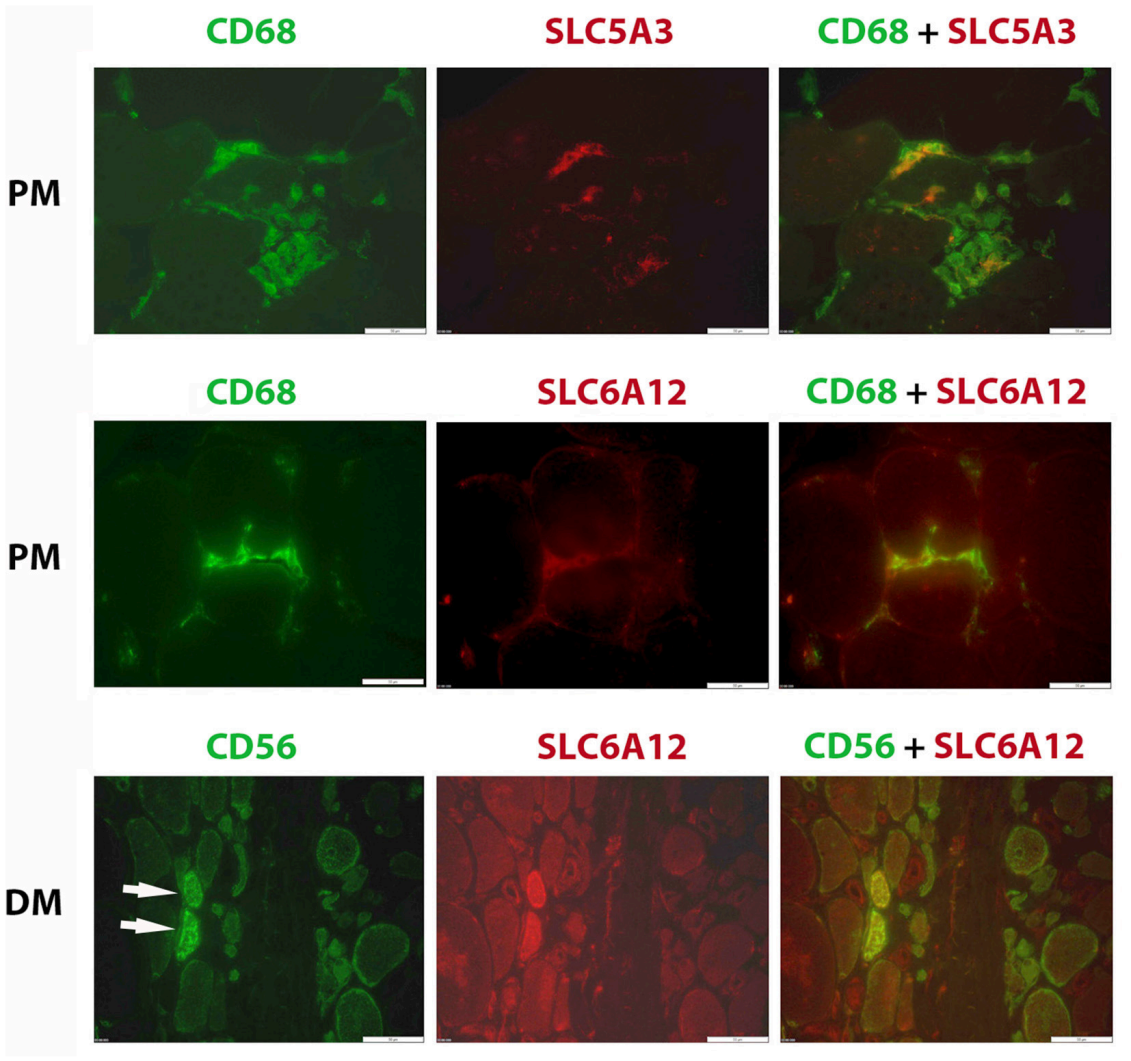
Immunofluorescent histostaining. Staining of SLC6A12 and AKR1B1 in muscle sections from patients diagnosed with inflammatory myopathy. Polymyositis (PM): A subset of CD68+ cells (AlexaFluor488, green), representing mostly M1 phenotype macrophages, express SLC5A3 and SLC6A12 (CY3, red). Dermatomyositis (DM): Two small regenerating muscle fibers (arrows), identified as strongly positive for CD56 (AlexaFluor488, green), express high levels of SLC6A12 protein (CY3, red). Scale bar =50 μm.

Confirming our earlier descriptive myopathological results on SLC5A3, SLC6A6, and AKR1B1 expression (24), we found these factors upregulated in regenerating muscle fibers present in biopsies in this new cohort of inflammatory myopathy patients. We now also add SLC6A12 to the regenerating muscle fiber's repertory of increased osmolyte accumulators (Figure 4). High osmolyte pathway member expression contrasted with the low levels present in muscle fibers in biopsies from healthy subjects. In tissues from muscular dystrophy patients, the SLC6A12 muscle fiber staining pattern was similar to inflammatory myopathies, with diffuse staining mostly in small regenerating CD56 positive muscle fibers and discontinuous sarcolemmal staining in subsets of CD56 negative muscle fibers (Supplementary Figure S3). We verified that this did not represent adjacent satellite cells and observed no co-localization with Pax3/7 staining.

SLC5A3 and SLC6A12 were expressed by inflammatory cells in inflammatory myopathy muscle biopsies (Figure 4). SLC6A12 was detected in subsets of the endomysial CD68+ cells surrounding muscle fibers in PM and IBM tissues. In comparison, only a small minority of tissue-infiltrating CD68+ cells in DM and in muscular dystrophy tissues were SLC6A12 positive, and the more sparse inflammatory cells observed in IMNM tissues were SLC6A12 negative. SLC6A12 was rarely observed in the CD8+ T-cells of PM and IBM tissues. SLC5A3 on the other hand, could be detected in a substantial part of CD3+ T-cells, with most non-invading CD8+ T-cells being negative while CD8+ cells actively invading nonnecrotic muscle fibers in PM and IBM were often strongly positive. The majority of CD68+ cells, and part of the CD206+ cells were also SLC5A3 positive. In contrast, inflammatory cells were invariably AKR1B1 and SLC6A6 negative in all muscle tissues tested.

## Discussion

Our earlier studies had identified osmolyte pathway members as biomarkers for inflammatory myopathies, yet the pathogenic routes behind their elevated expression remained unknown. The present study offers further context and allows us to speculate on the underlying mechanisms.

### Inflammatory stress induces osmolyte pathways in muscle cells

We here report that, in addition to increased NaCl concentrations, pro-inflammatory cytokines can induce osmolyte pathways in muscle cells in an *in vitro* setting. In response to osmotic stress, normal primary myotubes displayed a continuous increase of SLC5A3, SLC6A12, and AKR1B1 mRNA expression levels over time, while single cytokine pulse led to highest mRNA levels after 24 h, steadily decreasing afterwards and returning to near-normal levels at the 72 h time point. Immunocytochemical staining and western blotting experiments confirmed the transient induction pattern of SLC6A12 at the protein level. We cannot rule out fast cytokine degradation in the culture medium, yet this is unlikely as many have reported prolonged effects by single pulse cytokine treatments over several days (28–30). Using silencing techniques, we showed that compromising osmolyte accumulator expression negatively influenced cell growth only when muscle cells faced hyperosmotic conditions. We produced strongest evidence for AKR1B1, for which the knockdown regimen severely reduced AKR1B1 protein levels, yet did not compromise growth when myoblasts and myotubes were challenged with pro-inflammatory cytokines. Based upon this data, we speculate on different levels of importance of the osmolyte pathway dependent upon the challenges muscle cells face: osmolytes being essential protectors against osmotic stress while having a less vital yet regulatory role in the response to inflammatory stress. Our observations would need to be confirmed and analyzed further with knockdown combinations, as an important additional facet is the degree of redundancy of function displayed by the different osmolyte pathway members. In evidence, only SLC5A3 deficiency represents a lethal murine phenotype (31), with knockout of AKR1B1, SLC6A6, and SLC6A12 resulting in viable mice suffering from limited defects in renal function (32–34). The compensatory role of remaining osmolytes when activity of single pathway members is compromised, has long been confirmed in kidney cells (35).

Transcription factors NFκB and NFAT5 represent key regulators of cell's responses to inflammatory stress on the one hand and osmotic stress on the other hand. Not surprisingly therefore, pro-inflammatory cytokines were the most potent inducers of RelA, NFkB1, NFkB2 expression in muscle cells. However, we did observe some effects of NaCl-treatment on NFκB expression levels and, vice versa, NFAT5 expression responded to pro-inflammatory cytokines. Thus, muscle cells' responses to inflammatory and osmotic stress appear not separately regulated via NFκB and NFAT5, respectively.

The MAP kinases, a large family of Ser/Thr kinases that translate cell surface signals to the nucleus, play a crucial role in inflammation (36), which led us to study their involvement in muscle cells' responses to pro-inflammatory cytokines. We found Akt2 and HSP27 were most heavily phosphorylated in myotube cultures, but their activation was unaltered in response to inflammatory cytokines. Akt signaling influences muscle development and regeneration, affecting either initiation (Akt1) or maturation (Akt2) of myotubes (37). HSP27 is also associated with myotube differentiation and was found absent from myoblasts (38). The strong activation of Akt2 and HSP27 we observed in our *in vitro* model fits with the differentiated stage of the myotubes we studied in our experiments. The MAPKs of the p38 subgroup have been put forward as complex regulators of osmolyte pathways, through their hypertonicity-induced phosphorylation, and have been implicated in inflammatory disease (39). Our study found MAPK14 mRNA expression increased up to 2-fold by pro-inflammatory cytokines, and we observe increased phosphorylation of MAPK12 and MAPK13 in human primary myotubes treated for 24 h with IL1β+IFNγ. Of the extracellular signal-regulated kinases (ERK) on the other hand, we observed increased phosphorylation of MAPK3 (ERK1) in IL-1β+IFN-γ-treated myotubes. This is in line with our recent study that showed cytokines induce ERK1/2 phosphorylation, which subsequently leads to protein deposition and autophagy in muscle cell cultures (40). We propose that the 24 h response of muscle cells to pro-inflammatory cytokines may thus be regulated primarily via such phosphorylation-driven activation of residential MAPKs. Muscle cells exposed to osmotic stress for prolonged periods appear to require extra measures for their protection, culminating in 18-fold (MAPK14) and 9-fold (NFAT5) increases in expression levels.

### Osmolyte pathways are activated in regenerating muscle cells

Osmolytes are known regulators of skeletal muscle development, with the involvement of taurine and betaine already described in most detail. Taurine is essential for skeletal muscle buildup, and knockout mice lacking its transporter SLC6A6 display severe structural defects (41) and exercise intolerance (42). The trimethylglycine betaine appears also important for proper muscle functioning, although SLC6A12 knockout mice have been reported to develop only mild myopathy (34). Betaine promotes muscle fiber differentiation and myotube size (43) via stimulation of the mechanistic target of rapamycin pathway (44) and disturbed osmolyte balances have been implicated in muscle disease. Taurine levels were significantly reduced in muscle from myositis patients compared to healthy controls (45), while in urine on the other hand both taurine and betaine levels were increased in patients (46). In the murine Duchenne muscular dystrophy model, taurine content of muscle was low, mostly early in disease progression, and a reduction of its transporter SLC6A6 was observed (47). Yet, in the canine golden retriever muscular dystrophy model, muscle levels of taurine and SLC6A6 were 1.5- and 20-fold increased (48) compared to healthy dogs, pointing to possible differences between species and/or at different disease stages. Our localization studies revealed strong increases of osmolyte accumulators in small regenerating fibers. Possibly, protein replacement and refolding during the regeneration process are chaperoned by osmolytes, the latter aiding protection of functional protein conformations (49).

### Subsets of muscle-infiltrating inflammatory cells express SLC5A3 and SLC6A12

In addition to the muscle cells' general and dynamic repertory of osmolyte accumulators, we found two osmolyte pathway members, SLC5A3 and SLC6A12, selectively expressed in subsets of macrophages and T-cells infiltrating skeletal muscle. Interestingly, we observed an association with active invasion of nonnecrotic muscle fibers, a phenomenon typically observed in PM and IBM muscle tissues (50), which fits with current notions of how osmolytes behave as potent cytotoxic regulators. Cytotoxic macrophages have been shown to accumulate betaine, myoinositol and taurine as compatible organic osmolytes in response to osmotic stress, via MAPK-regulated upregulation of osmolyte transporters (51, 52). It is well known that macrophages have versatile functionalities, with a spectrum stretching out between the classical inflammatory M1 phenotype, to the alternative anti-inflammatory, tissue repair-oriented M2 phenotype (53). Significantly higher levels of AKR1B1 mRNA and protein have been reported in M1 macrophages compared with M2-polarized macrophages (54). The upstream transcription factor NFAT5 is enhanced by the M1-promoting pro-inflammatory and hypoxic conditions associated with autoimmune diseases, which is particularly suggested to regulate the chemokine MCP-1 and subsequent synovial macrophage survival in rheumatoid arthritis (55). We found a subset of CD68+ cells, regarded as representing mostly M1 macrophages, to express SLC5A3 and SLC6A12, most prominently in PM and IBM tissues. In addition to macrophages, T-cells have also been shown to engage NFAT5-regulated pathways in their development and activation (56) and, in accordance, we found SLC5A3 and SLC6A12 expression in a minority of T-cells. In helper T-cells (Th-cells), salt-induced NFAT5 activity promotes their differentiation into Interleukin 17-producing Th-cells (Th17-cells) (57). This is relevant to inflammatory myopathy, as IL-17 induces and maintains chronic inflammation, and Th17-cells represent a pathogenic subset of T-cells associated with inflammatory myopathy (58).

## Conclusions

The expression of osmolyte pathway members extends to human tissues that normally are not exposed to hypertonicity, which has led to the assumption that these factors have additional more versatile functions. In this respect, they have been put forward as biomarkers for inflammation (59) and tumor metastasis (60). The data we presented here points to a general role for osmolyte accumulation, via AKR1B1, SLC5A3, SLC6A6, and SLC6A12 upregulation, in muscle cells challenged by inflammatory stress, presumably in an attempt to stabilize protein function in sight of the changed proteome during regeneration. In addition, our data suggest an individual inflammatory role for SLC5A3 and SLC6A12 as potential regulators of the myocytotoxicity displayed by muscle tissue-infiltrating auto-aggressive immune cells. The data we offer further adds to the complexity of inflammatory myopathy immunopathogeneses, broadening our understanding of this heterogeneous group of diseases.

## Author contributions

BD conceived, designed, and executed the study. JW and JD analyzed patient data and material. BD, JZ, and TŠ carried out experiments and analyzed the data. BD drafted the manuscript, which was critically revised by JZ, JS, and JD.

### Conflict of interest statement

The authors declare that the research was conducted in the absence of any commercial or financial relationships that could be construed as a potential conflict of interest.

## Abbreviations

AKR1B1: aldose reductase
DM: dermatomyositis
dMyHC: developmental myosin heavy chain
ERK: extracellular signal-regulated kinase
GAPDH: glyceraldehyde-3-phosphate dehydrogenase
GSK-3α/β: glycogen synthase kinase 3α/β
HSP70: heat shock protein family of 70kd
IBM: sporadic inclusion body myositis
IFNγ: Interferon γ
IL1β: Interleukin 1β
IMNM: immune-mediated necrotizing myopathy
iNOS: inducible nitric oxide synthase
LTβ: lymphotoxin β
MAPK: mitogen-activated protein kinase
MCP-1: monocyte chemo-attractant protein-1
MSK2: mitogen- and stress-activated kinase 2
NFAT5: nuclear factor of activated T-cells 5
NFκB: nuclear factor κB
PM: polymyositis
siRNA: silencing RNA
SLC5A3: solute carrier sodium-myoinositol cotransporter
SLC6A6: solute carrier taurine transporter
SLC6A12: solute carrier betaine and γ-amino-n-butyric acid (GABA) transporter
Th17: Interleukin 17-producing T-cells
TNFα: tumor necrosis factor α.
