# Supplementary figures and images for: Induction of Osmolyte Pathways in Skeletal Muscle Inflammation: Novel Biomarkers for Myositis
Source: Front Neurol. 2018 Oct 11;9:846. doi: 10.3389/fneur.2018.00846 (PMC6193116; doi:10.3389/fneur.2018.00846)

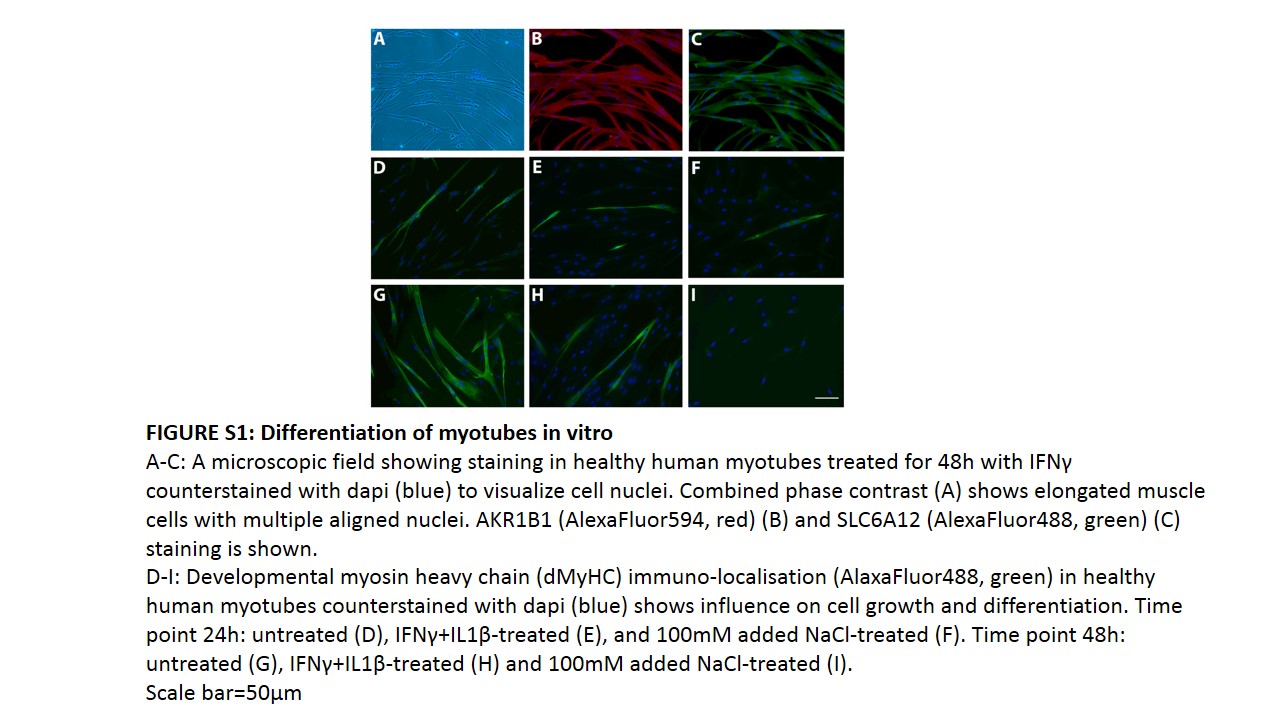

Supplement: Supplementary file 1 [file Image_1.jpg]

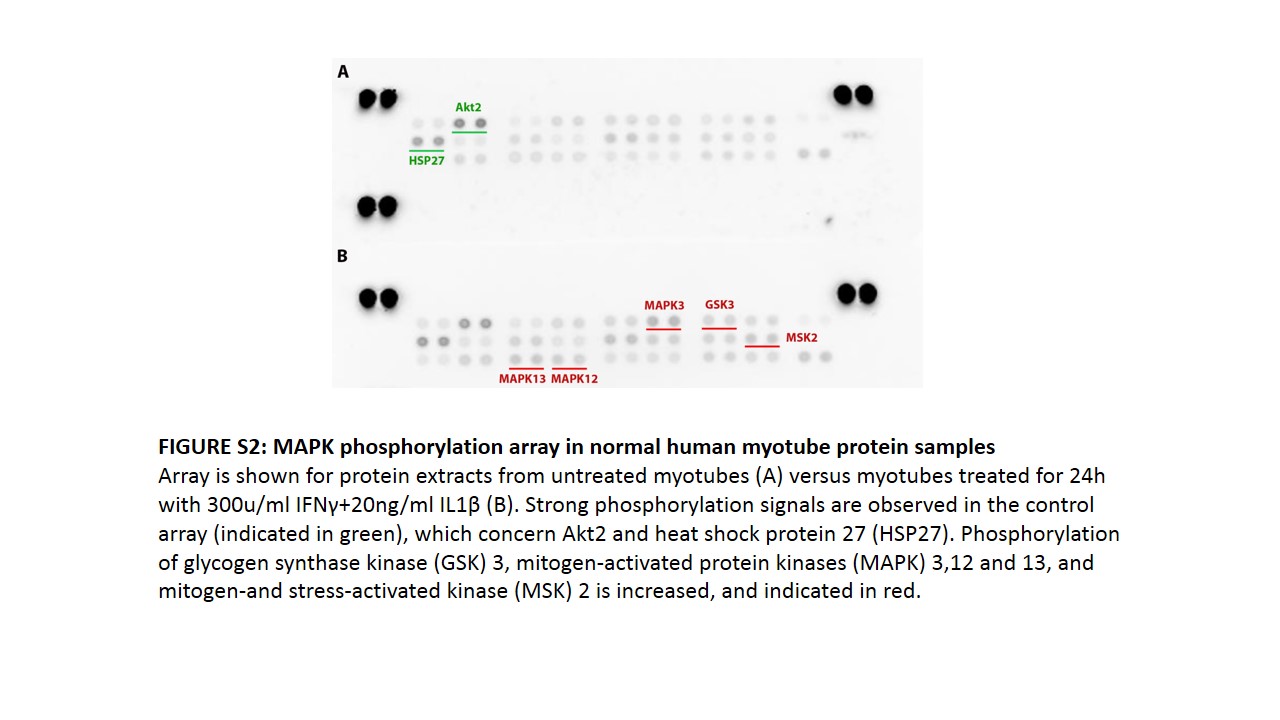

Supplement: Supplementary file 2 [file Image_2.jpg]

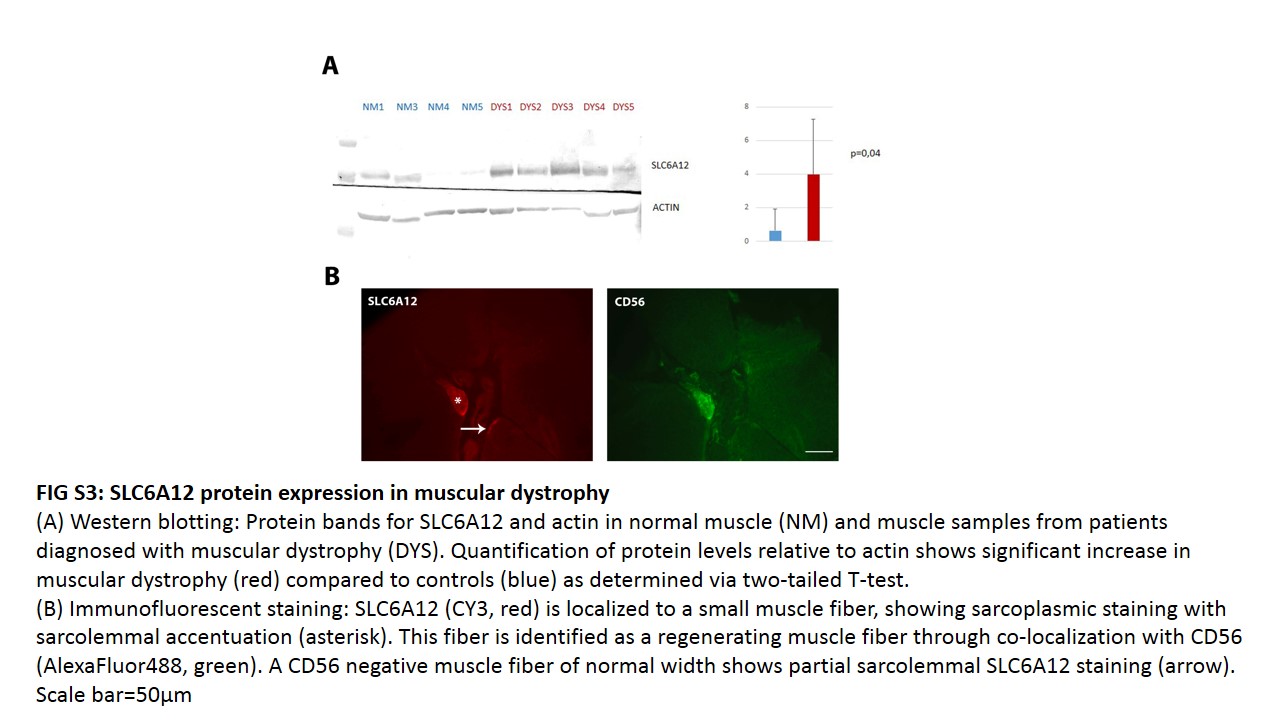

Supplement: Supplementary file 3 [file Image_3.jpg]
