# Supplementary material for: Induction of Osmolyte Pathways in Skeletal Muscle Inflammation: Novel Biomarkers for Myositis
Source: Front Neurol. 2018 Oct 11;9:846. doi: 10.3389/fneur.2018.00846 (PMC6193116; doi:10.3389/fneur.2018.00846)
Supplement: Supplementary file 4 [file Data_Sheet_1.docx]

**Table S1: Pathological information of muscular dystrophy patients**

|  | **gender** | **age** | **myopathology** | **genetic alteration** |
| --- | --- | --- | --- | --- |
| **DYS1** | F | 63 | mild muscular dystrophy, no apparent necrotic fibers nor inflammation | unknown |
| **DYS2** | F | 64 | active chronic muscular dystrophy, few necrotic fibers | unknown |
| **DYS3** | F | 40 | muscular dystrophy, typical inclusion body myositis biopsy with nonnecrotic invaded muscle fibers and rimmed vacuoles | valosin containing protein *VCP* c.374G>A (p.Gly125Asp) causing hereditary inclusion body myositis |
| **DYS4** | M | 31 | active muscular dystrophy, inflammation restricted to necrotic fibers | unknown |
| **DYS5** | F | 23 | mild muscular dystrophy, inflammation restricted to necrotic fibers | fukutin-related protein *FKRP* c.1100T>C (p.Ile367Thr) and c.826C>A (p.Leu276Ile) causing limb girdle muscular dystrophy type 2I |
| **DYS6** | F | 27 | active muscular dystrophy, few necrotic fibers | fukutin-related protein *FKRP* c.826C>A (p.Leu276Ile) causing limb girdle muscular dystrophy type 2I |
| **DYS7** | M | 3 | severe muscular dystrophy, mild inflammation | dystrophin *DYS* exon 65 c.9527A>G alternative splice donor site causing Duchenne muscular dystrophy |
| **DYS8** | M | 8 | severe muscular dystrophy, mild inflammation | dystrophin *DYS* exon 63 c.9470delA frameshift mutation causing Duchenne muscular dystrophy |
